# High frequency of enterovirus D68 in children hospitalised with respiratory illness in Norway, autumn 2014

**DOI:** 10.1111/irv.12300

**Published:** 2014-12-23

**Authors:** Karoline Bragstad, Kirsti Jakobsen, Astrid E Rojahn, Marius K Skram, Kirsti Vainio, Mona Holberg-Petersen, Olav Hungnes, Susanne G Dudman, Anne-Marte B Kran

**Affiliations:** a Department of Virology, Norwegian Institute of Public HealthOslo, Norway; b Department of Microbiology, Oslo University HospitalOslo, Norway; c Department of Pediatrics, Oslo University HospitalOslo, Norway; d University of Oslo, Institute of Clinical MedicineOslo, Norway

**Keywords:** Acute flaccid paralysis, enterovirus D68, picornavirus, respiratory infections, respiratory viruses

## Abstract

**Objectives:**

An unexpectedly high proportion of children were admitted for severe respiratory infections at the Oslo University Hospital, Ullevål, Norway, during September and October, 2014. In light of the ongoing outbreak of enterovirus-D68 (EV-D68) in North America a real-time RT-PCR for screening of enterovirus and enterovirus D68 was established.

**Design:**

We developed a duplex real-time RT-PCR for rapid screening of enterovirus D68. The method target the 5′ non-translated region (NTR) of the HEV genome at a location generally used for enterovirus detection.

**Sample:**

Nasopharyngeal samples (*n* = 354), from children <15 years of age, received for respiratory virus analysis in OUH during September 1st and October 31nd, 2014, were tested for enterovirus and screened for enterovirus D68.

**Main outcome measures and results:**

The duplex real-time RT-PCR method was an efficient tool for rapid screening for EV-D68 in respiratory specimens. Enterovirus was detected in 66 (22%) of 303 pediatric nasopharyngeal samples collected from children hospitalised with acute respiratory infection within the two-month period. Out of these, 33 (50%) were EV-D68. EV-D68 was associated with acute flaccid paralysis in one child.

**Conclusions:**

An unexpectedly high proportion of children admitted for severe respiratory infections at the Oslo University Hospital, Ullevål, Norway, were diagnosed with EV- D68 during September 1st and October 31nd, 2014. These results emphasise that greater vigilance is required throughout Europe as enteroviruses are cause of severe respiratory disease.

## Introduction

EV-D68 was first described in 1962^1^ and has been sporadically detected up to 2008. EV-D68 is one of the four serotypes (-D70, -D94, -D111 and -D68) assigned to human enterovirus (HEV)-D. Several outbreaks have occurred in recent years, and the largest outbreak is currently ongoing in North America.^2^ Over the last several months (August to November 2014), EV-D68 has been associated with severe respiratory illness and comprises 40% of enterovirus-positive respiratory samples in North America (CDC, 2014). Also in the Netherlands, EV-D68 continues to circulate in a seasonal pattern after an outbreak in 2010.^3^ Enteroviruses cause a broad variety of illnesses, mild to severe, mainly in children (e.g. hand-foot-and-mouth disease, meningitis and encephalitis, febrile exanthematous illness and conjunctivitis). Non-polio enteroviruses have also been associated with acute flaccid paralysis (AFP).^4^,^5^

EV-D68 has rarely been detected in Norway since the surveillance of enteroviruses started in 1965. Respiratory samples are only occasionally investigated for enteroviruses in hospitals, and samples received for surveillance of respiratory illness are mainly tested by a generic enterovirus PCR not distinguishing the different serotypes. However, enteroviruses from stool samples are typed by neutralisation test using antisera (monovalent and pools) supplied by WHO as part of the AFP surveillance.

In September 2014, the Oslo University Hospital (OUH) noticed an increased number of children with severe respiratory symptoms admitted to the hospital. Due to the recent outbreak of EV-D68 in America, nasopharyngeal samples from these patients were tested specifically for this virus, rendering a high proportion of EV-D68. Subsequently, nasopharyngeal samples from all children <15 years in OUH, hospitalised with respiratory illness from 1 September 2014 to 31 October 2014, were screened for enterovirus. By applying a highly sensitive duplex real-time RT-PCR distinguishing between enteroviruses and D68 (including EV-D viruses and polio), an ongoing large outbreak with EV-D68 in the Oslo area was effectively identified.

## Methods

### Duplex enterovirus One-Step RT-PCR assay

We developed a duplex real-time RT-PCR for rapid screening of enterovirus D68. The method targets the 5′ non-translated region (NTR) of the enterovirus genome at a location generally used for detection. The generic enterovirus primers and probes are updated modifications of the method developed by Brittain-Long *et al*.^6^ and Lars P. Nielsen (Statens Serum Institut, Copenhagen, Denmark). A probe for detection of enteroviruses in group D, particularly D68, and polio was included (Table1). The probe was selected based on *in-silico* analysis of all sequences in GenBank covering the exact length of the primer binding region (100% coverage, >91% nucleotide identity and a score of >200, *n* = 164). Primers and probe matches towards D68 viruses were confirmed with alignment of all D68 viruses available in GenBank for that region (*n* = 113). *In-silico* specificity of the probe was confirmed by BLAST search of the probe sequence, indicating match also towards polio 1, 2 and 3 virus and enterovirus 70 and 64 in group D. Match with other enteroviruses in the D-group can not be excluded. The method can be used for highly sensitive first-line screening for EV-D68, but additional verification of positives by alternative D68-specific real-time PCR assays or sequencing is advisable.

**Table 1:** Duplex EV-EVD68 real-time RT-PCR primers and probes*

Assay	Detection	Target region	Forward	Reverse	Probe
Duplex EV-EVD68	Generic enterovirus	5′ NTR	GGTGYGAAGAGTCTATTGAGC	CACCCAAAGTAGTCGG	FAM-CCGGCCCCTGAATG-BHQ1
	EV-D68 (EV-D/Polio)				ROX-CGCAAGTCCGTGGCGGAA-BHQ1

* Final primer and probe concentrations were 400 nm primer and 80 nm probe.

Nucleic acid from clinical samples (200 μl) was extracted on a MagNA Pure LC Instrument applying the MagNa Pure LC Total Nucleic Acid Isolation Kit (Roche diagnostics, Basel, Switzerland). The RT-PCR reactions were performed with the oligonucleotide primer and probe sequences given in Table1 and amplified by the OneStep RT-PCR Kit (QIAGEN, Hilden, Germany) on the RotorGene cycler system (Corbett/QIAGEN) applying 20 minutes at 50°C, 15 minutes at 95°C, followed by 45 cycles of 15 seconds at 95°C and 60 seconds at 55°C.

**Figure 1:**
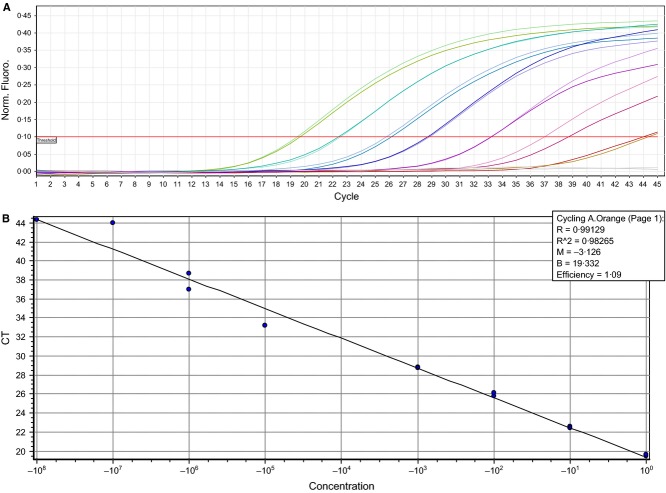
Fluorescents diagram (A) showing detection sensitivity of EV-D68 and standard curve (B) with 10-fold dilution series of EV-D68 RNA in duplicates (undiluted to 10^−8^) detected by the EV-D68 probe in the duplex EV-EVD68 real-time RT-PCR assay.

The duplex EV-EVD68 assay was verified by investigating the specificity applying the QCMD Enterovirus 2014 EQA panel [QAV984104 (EVRNA14)]. All samples were typed correctly, detecting the enteroviruses (Coxackievirus A9, A16, B5, A24, B3, echovirus 11, enterovirus 71) in the FAM-channel, and a specific ROX signal was only seen for the single EV-D68 sample. Cross-reaction with rhinovirus-positive clinical samples (*n* = 14) has not been experienced. The specificity was further examined by running the assay on 54 clinical samples where the EV-D68-positive samples had been verified by sequencing (the ‘gold-standard’ assay) and/or by a real-time RT-PCR assay specific for EV-D68^7^ (The University Medical Center Groningen, The Netherlands). The positive predictive value for the duplex assay detection for EV-D68 was 96% (one sample was suspected to be a double infection with rhino and EV-D68), negative predictive value was 100%, sensitivity 100% and specificity 96%. The assay had high sensitivity; allowing a EV-D68-positive clinical sample of ROX-Ct of 19·6 to be diluted 10-fold, down to a 1/10 million dilution, and still be detected at Ct 44·2 and 44·3 in FAM and ROX, respectively (Figure1). The assay had a reaction efficiency for enterovirus detection (FAM) of 1·11 (*R* = 0·995, slope = −3·086) and 1·09 for EV-D68 detection (ROX) (*R* = 0,991, slope = −3,126). Variations in *C*_t_ values of run controls between three replicate runs were of 0·26%, (0·05 standard deviation) indicating a robust real-time RT-PCR assay with high precision.

## Results and discussion

As an initial screen, all nasopharyngeal samples from children <15 years of age, received for respiratory virus analysis in OUH during 1 September 2014 and 31 October 2014, were tested for enterovirus. Enterovirus was detected in 66 (22%) of 303 samples from children hospitalised with acute respiratory infection, and in five (10%) of 51 samples received from outpatient clinics. EV-D68 was verified in 33 (50%) of the enterovirus-positive samples from hospitalised patients, and in one outpatient (20%) (Table2).

**Table 2:** Detections in Oslo University Hospital of enterovirus and EV-D68 in respiratory samples from children <15 years old, 1 September 2014 to 31 October 2014

	Number of clinical specimens tested	Number of enterovirus positives (% of specimens tested)	Number of EV-D68-positive specimens (% of enterovirus-positive specimens
Outpatients	51	5 (10)	1 (20)
Hospitalised	303	66 (22)	33 (50)

There have been no deaths associated with the enterovirus infections. None of the children with merely respiratory symptoms were admitted to intensive care units (ICU), but 30 patients were hospitalised for a few hours up to 5 days. Seven of these were given extra oxygen. Median days of hospitalisation were 2 days. Among the hospitalised children with EV-D68, 18 (54%) had severe respiratory symptoms. Most of these (*n* = 16, 89%) had a history of asthma, allergy, atopic eczema or other chronical pulmonary diseases. One previously healthy child with EV-D68 had symptoms of neurological disease or paralysis, like AFP. This patient was admitted to the intensive care unit on September 22nd and is still (December 4th) given intensive care. Sixty-six (93%) of the patients with verified enterovirus were of age 1 month to 6 years. However, while 27 patients (73%) with enterovirus other than EV-D68 were <2 years, EV-D68 was equally detected in all ages <7 years (Table3).

**Table 3:** Age distribution of patients with EV-D68 (outpatients and hospitalised) and other enteroviruses, Oslo, 1 September 2014 to 31 October 2014

Age	EV-D68	EV (other than D68)
0	6 (18%)	17 (46%)
1	5 (15%)	10 (27%)
2	6 (18%)	2 (5·4%)
3	4 (12%)	1 (2·7%)
4	4 (12%)	3 (8·1%)
5	3 (8·8%)	0 (0%)
6	4 (12%)	1 (2·7%)
>6	2 (5·9%)	3 (8·1%)
Total	34	37

To get a first estimate of EV-D68 prevalence in the community, samples submitted by general practitioners nationwide to the National Influenza Centre for WHO in Norway (NIC-Norway), NIPH, Oslo, were screened for EV-D68. These were sentinel samples from patients <25 years, not diagnosed with influenza (*n* = 45, 0–4 years = 3, 5–14 years = 8, 15–24 years = 34), received for surveillance for influenza and other respiratory viruses season 2013–14. Enterovirus was detected in three of 45 (6·7%) sentinel samples (two in age group 5–14 and one in age group 15–24). None of these were EV-D68 positives. In addition, the 14 most recent (all age groups) of 41 respiratory samples, which had tested positive for rhinovirus, were screened for EV-D68. One case of EV-D68 (27 year old woman, sampled October 2014) was detected and verified by sequencing.

The Netherlands observe a higher incidence of EV-D68 cases in their ILI/ARI surveillance compared to the enterovirus surveillance.^3^ About 1·6% of all ILI/ARI specimens were positive for enterovirus, and 38% of these were EV-D68 positives. In comparison, we have so far only tested 59 sentinel samples in total, four (6·8%) were enterovirus positives and one (25%) of these was EV-D68 positive. As in China,^8^ we found EV-D68 to be one of the most common enterovirus (50%) in patients with severe acute respiratory infection (SARI). The prevalence of EV-D68 in children with SARI has been ranging between 0·32% and 12·37% according to CDC (CDC, 2011) and is currently at about 40% of all tested samples (mainly children) in the United States (CDC, 2014). This initial study indicates a prevalence of EV-68 in 9·6% (34/354) (Table2) of hospitalised children in the Oslo area, September/October 2014.

Although regarded as a virus especially infecting children <5 years of age, high prevalence in adults has been reported,^8^,^9^ together with pre-existing antibodies in most adults towards EV-D68.^10^ Low rates of EV-D68 in sentinel samples might however reflect the few sentinel samples submitted coming from children. In our study, the only EV-D68-positive sentinel sample was found in a 27 year old and in addition, we found EV-D68 equally distributed among all age groups between 1 month and 6 years of age, compared with the high fraction of patients <2 years with other enteroviruses. This supports that EV-D68 can be found in many age groups, not only very young children. Further analysis of all age groups of both outpatients and hospitalised patients in Norway has to be undertaken to complete the picture.

Human enterovirus infections seem to circulate in a seasonal pattern^3^ during the autumn and early winter in the Northern Hemisphere, and epidemics occur every 4–5 years.^8^ The most recent seems to be occurring in northern America 2014. Currently, several distinct clades have emerged and circulate globally.^11^ Our report indicates that EV-D68 is prevalent also in Europe and in November, one additional, previously heathy child in the Oslo region with AFP was confirmed infected with EV-D68. Therefore, greater vigilance should be given to enterovirus infections of the respiratory tract and as cause of AFP. Rapid, specific and sensitive screening methods should be in place. We have demonstrated that the RT-PCR assay presented can be a useful tool for rapid and efficient identification of a local outbreak of EV-D68.

Further analyses are ongoing to gather a comprehensive clinical and virological overview of the situation in Norway together with in-depth analysis of the viruses circulating in comparison with the viruses causing AFP.

## Authors' contributions

Karoline Bragstad developed and validated the duplex EV-EVD68 assay, tested the initial samples, wrote the first draft and finalised the manuscript. Olav Hungnes provided sentinel specimens, evaluated the assay and contributed with valuable suggestions. Kirsti Vainio supplied the QCMD panel with results together with cultivated D68 virus for controls. Marius K. Skram suggested testing of the first EV-D68-positive samples and has since contributed in including patients. Mona Holberg-Petersen developed the enterovirus RT-PCR used in routine diagnostics at OUH and was responsible for sequencing and verification of E-D68 at OUH. Astrid Rojahn was responsible for collecting clinical data and coordinating the collaboration between the clinic and laboratory. She was also responsible for clinical follow-up and treatment of the hospitalised patients. Kirsti Jakobsen performed screening for enterovirus. Anne-Marte Bakken Kran initiated the collaboration and testing of samples together with Susanne G. Dudman, contributed to study design and collection of data and performed analyses and interpretation of data together with Kirsti Jakobsen. All authors reviewed the manuscript critically and supplemented data and text to the manuscript.
